# Supplementary material for: Heat shock protein DNAJA2 controls insulin signaling and glucose homeostasis by preventing spontaneous insulin receptor endocytosis
Source: Nat Commun. 2025 Nov 13;16:9973. doi: 10.1038/s41467-025-64948-0 (PMC12615670; doi:10.1038/s41467-025-64948-0)
Supplement: Supplementary file 1 — Supplementary information [file 41467_2025_64948_MOESM1_ESM.pdf]

Supplementary Information

Heat shock protein DNAJA2 controls insulin signaling and glucose homeostasis by preventing spontaneous insulin receptor endocytosis

Yuanhua Qin[1,2,#], Wenjun Wu[1,#], Kequan Lin[3], Anthony J. Davis[4] and Yaping Huang[1,2,*]

[1]Institue for Molecular and Cellular Therapeutics, Chinese Institutes for Medical Research, Beijing, China 100069

[2]School of Basic Medical Sciences, Capital Medical University, Beijing, China 100069

[3]Department of Cardiology of the Second Affiliated Hospital, Zhejiang University School of Medicine, Hangzhou, China 310009

[4]Department of Radiation Oncology, University of Texas Southwestern Medical Center, Dallas, TX, USA 75390

[#]Contribute equally

***Correspondence to:**

Yaping Huang (huangyp@cimrbj.ac.cn)

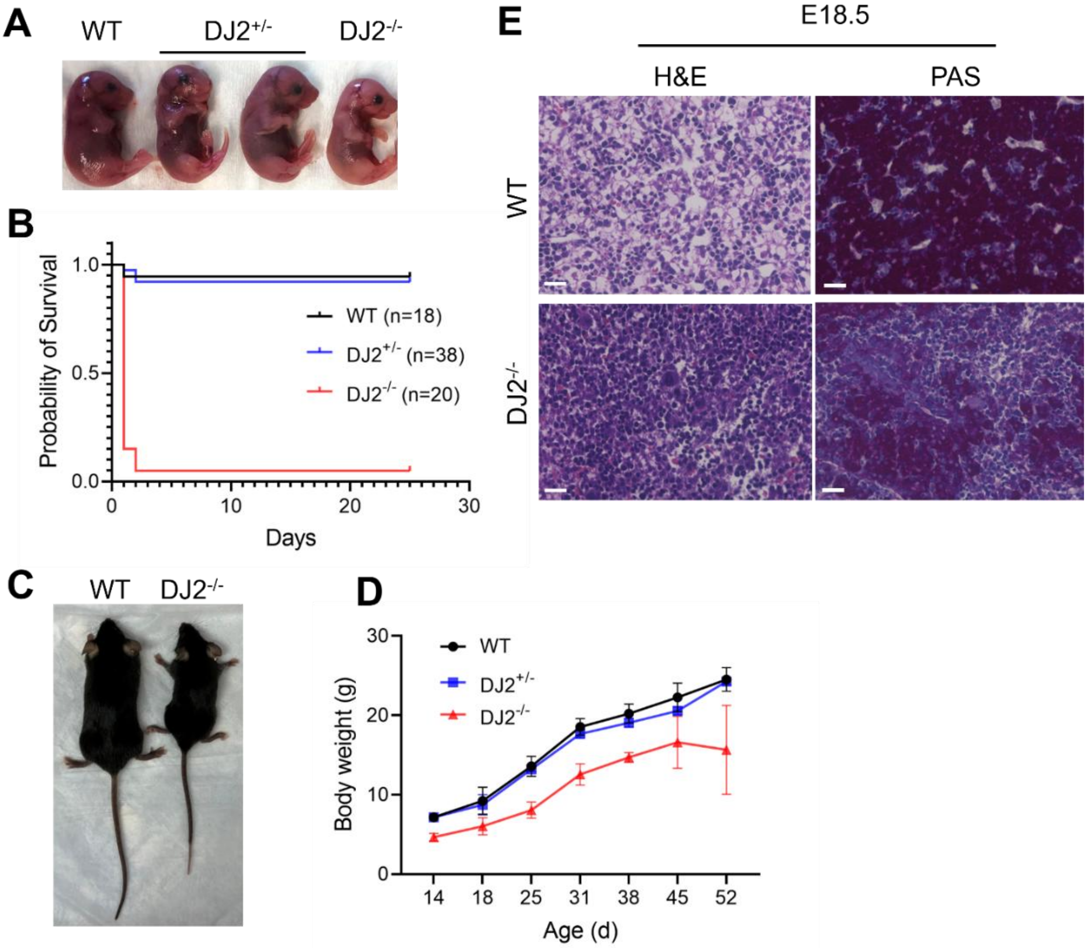

Supplementary Figure 1. Neonatal lethality and growth retardation of DJ2$^{-/-}$ mice, related to Figure 1. (A) Morphologies of WT, DJ2$^{+/-}$ and DJ2$^{-/-}$ embyos at E18.5. (B) Surviving curves of WT, DJ2$^{+/-}$ and DJ2$^{-/-}$ newborns. (C) Morphologies of WT and DJ2$^{-/-}$ adult mice. (D) Growth curves of WT (n = 5), DJ2$^{+/-}$ (n = 3) and survived DJ2$^{-/-}$ (n = 4) mice. (E) Representative images of H&E staining and PAS staining of livers from WT and DJ2$^{-/-}$ embryos at E18.5. *n* = 2 experimental repeats. Scale bar, 100 μm. Source data are provided as a Source Data file.

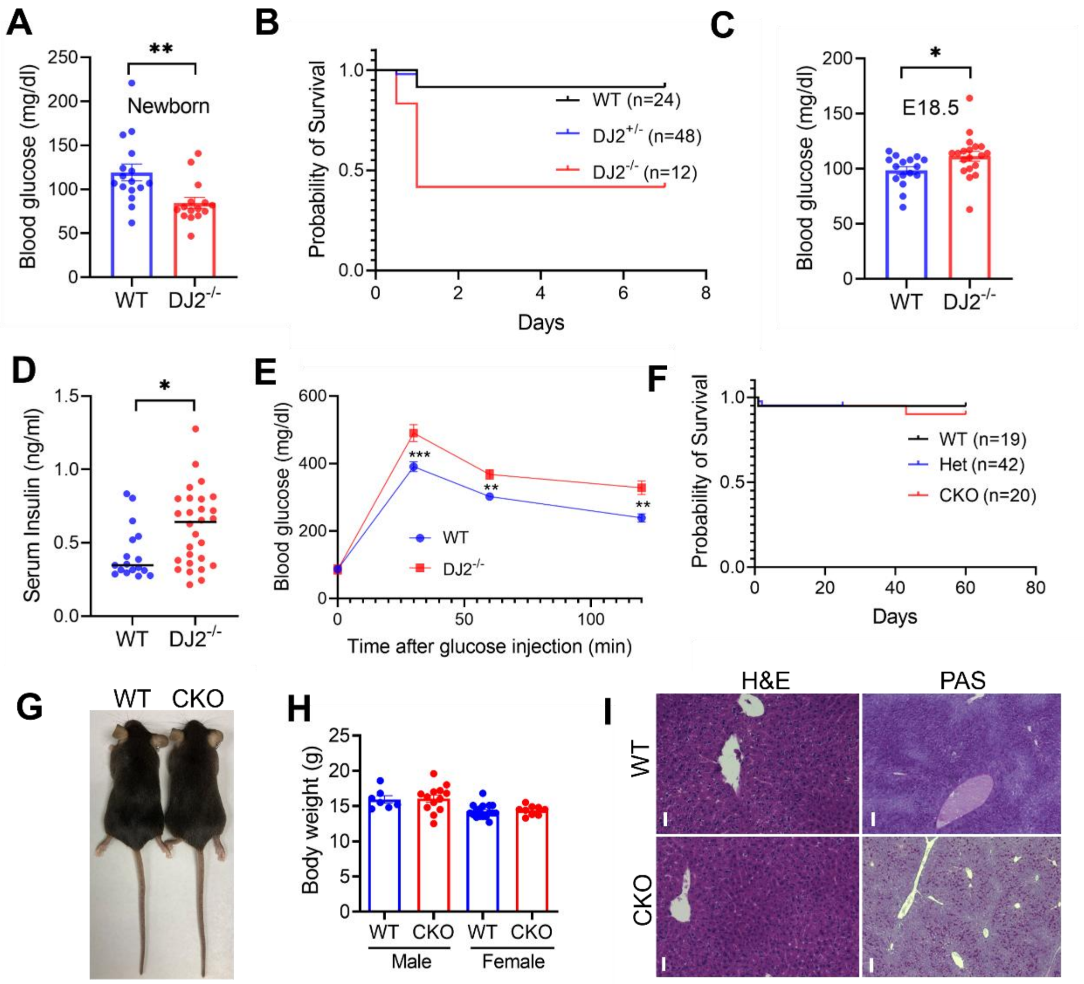

Supplementary Figure 2. DNAJA2 deficiency disrupts glucose homeostasis, related to Figure 1. (A) Blood glucose levels of WT (n = 16) and DJ2$^{-/-}$ (n = 15) newborns. (B) Surviving curves of WT, DJ2$^{+/-}$ and DJ2$^{-/-}$ newborns injected with glucose during the first 24 h after birth. (C-D) Blood glucose levels (C) and serum insulin levels (D) of cesarean sectioned WT and DJ2$^{-/-}$ embryos at E18.5 when the dams are in fed status. (E) Glucose tolerance test in WT and DJ2$^{-/-}$ embryos. (F) Surviving curves of WT, heterozygous (Het) and liver-specific *DNAJA2*-KO (CKO) mice. (G) Morphologies of WT and CKO adult mice. (H) Fasting body weight of WT and CKO mice. (I) Representative images of H&E staining and PAS staining of livers from 2-month-old WT and CKO mice. *n* = 2 experimental repeats. Scale bar, 100 μm. Data are shown as means ± SEM (A, C and E) or median (D). P values were determined by two-tailed unpaired t test with Welch's correction. *p<0.05; **p<0.01; ***p<0.001. Source data are provided as a Source Data file.

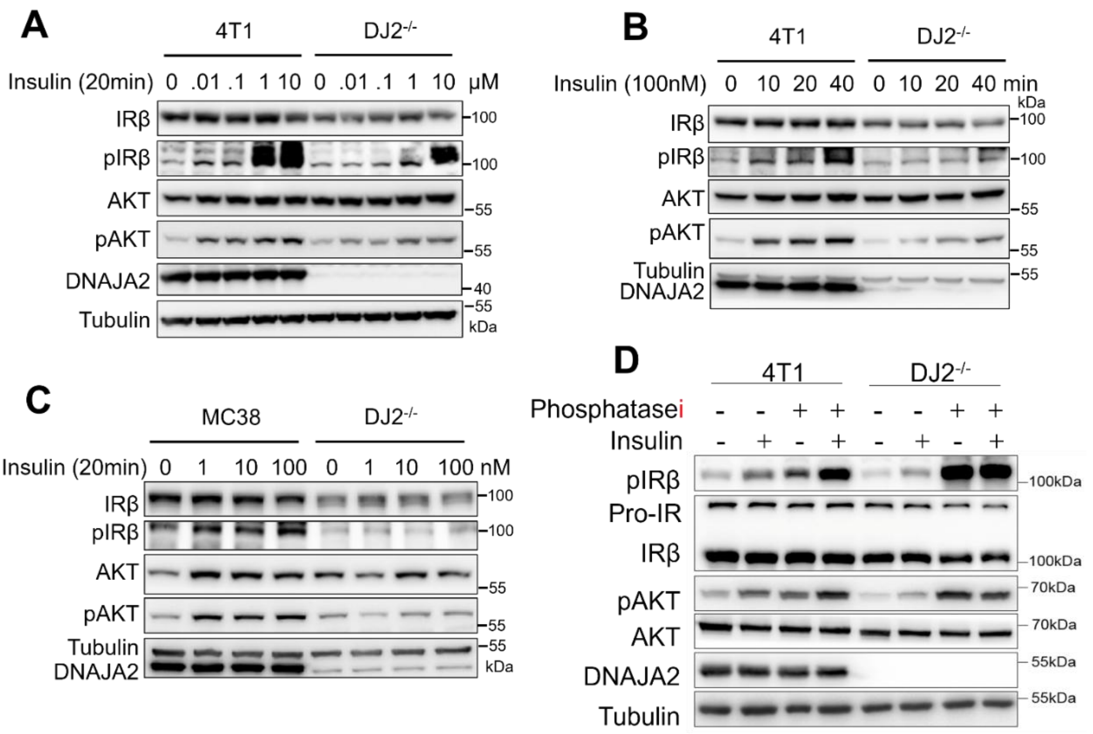

Supplementary Figure 3. DNAJA2 deficiency impairs insulin signaling in tumor cell lines, related to Figure 2. (A) Immunoblot analyses of pIRβ and pAKT in WT and DJ2$^{-/-}$ 4T1 cells treated with the indicated concentrations of insulin for 20 min. *n* = 3 experimental repeats. (B) Immunoblot analyses of pIRβ and pAKT in WT and DJ2$^{-/-}$ 4T1 cells treated with 100 nM insulin for a time course as indicated. *n* = 3 experimental repeats. (C) Immunoblot analyses of pIRβ and pAKT in WT and DJ2$^{-/-}$ MC38 cells treated with the indicated concentrations of insulin for 20 min. *n* = 2 experimental repeats. (D) Immunoblot analyses of pIRβ and pAKT in WT and DJ2$^{-/-}$ 4T1 cells treated with 100 nM insulin for 20 min in the absence or prescence of pan-phosphatase inhibitor pre-treated for 5 h. *n* = 4 experimental repeats. Source data are provided as a Source Data file.

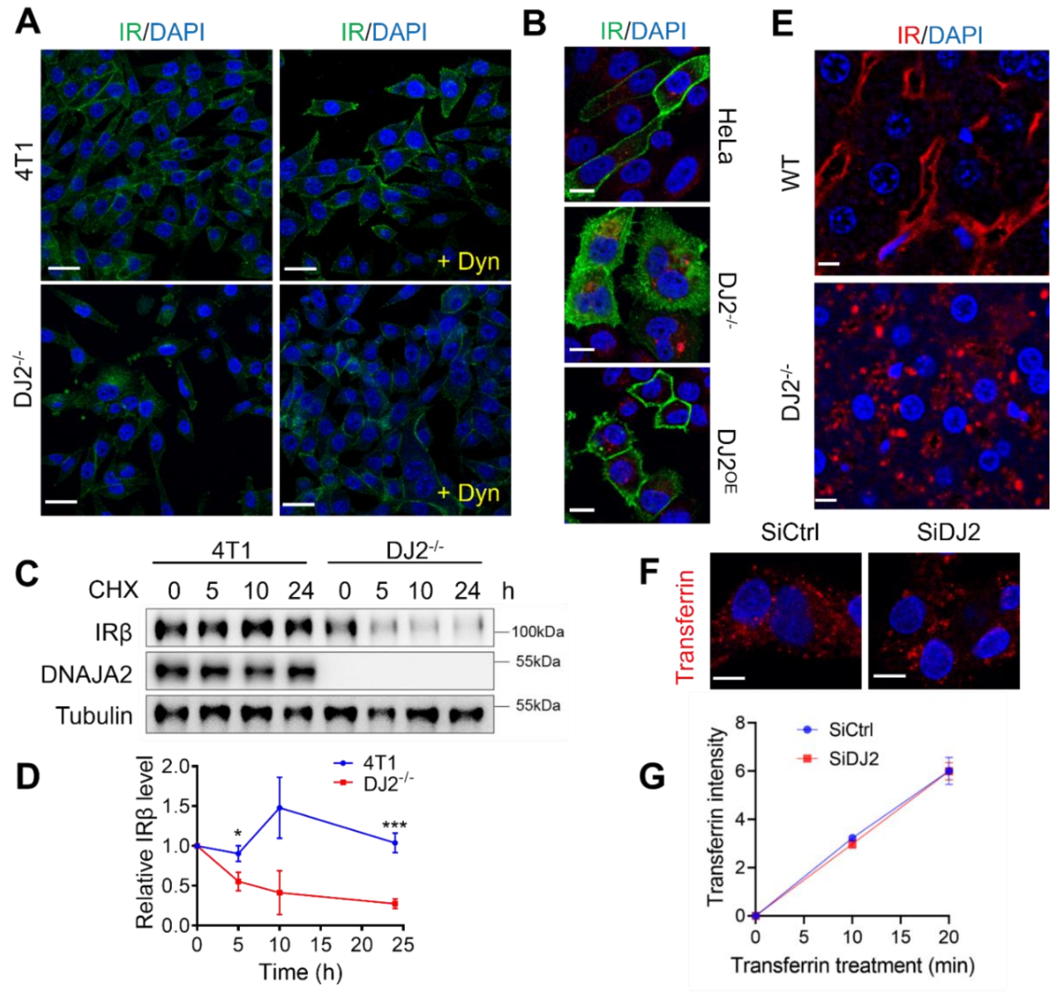

Supplementary Figure 4. DNAJA2 inhibits IR endocytosis, related to Figure 3. (A) Immunoflourescence analysis of the subcellular localization of IR in WT and DJ2$^{-/-}$ 4T1 cells treated with or without 50 μM dynasore. Scale bar, 50 μm. *n* = 3 experimental repeats. (B) Immunoflourescence analysis of the subcellular localization of ectopically expressed IR in WT, DJ2$^{-/-}$ and DJ2^{OE} HeLa cells starved for 15 h. *n* = 3 experimental repeats. Scale bar, 10 μm. (C) Immunoblot analyses of the protein half-life of IRβ in WT and DJ2$^{-/-}$ 4T1 cells. Cells were treated with 50 μg/ml Cycloheximide (CHX) for the indicated times and harvested for western blot assay. (D) Quantifications of relative IRβ protein levels in WT and DJ2$^{-/-}$ 4T1 cells. (E) Representative images showing IR and DAPI staining in liver sections of 2-month-old WT and survived DJ2$^{-/-}$ mice. *n* = 2 experimental repeats. Scale bar, 20 μm. (F) Representative images showing the intensities of Alexa-568-labeled transferrin after 20-min treatment in SiCtrl and SiDJ2 HepG2 cells. (G) Quantifications of Alexa-568-labeled transferrin intensities as shown in F. Data are shown as means ± SEM.

P values were determined by two-tailed unpaired t test with Welch's correction. *p<0.05; ***p<0.001. Source data are provided as a Source Data file.

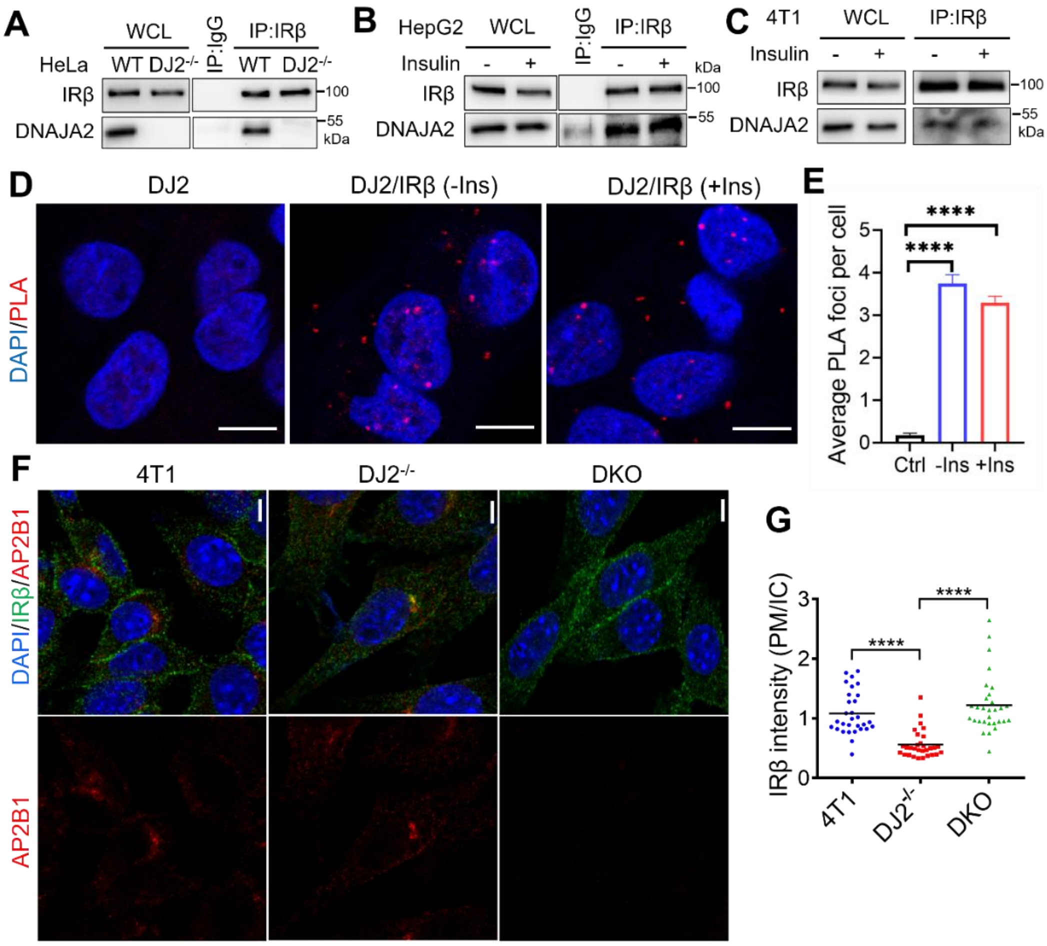

Supplementary Figure 5. DNAJA2 interacts with IRβ and inhibits its endocytosis under basal state, related to Figure 4. (A) Co-IP assay showing the interaction between IRβ and DNAJA2 in WT and DJ2$^{-/-}$ HeLa cells. *n* = 2 experimental repeats. (B-C) Co-IP assays showing the interaction between IRβ and DNAJA2 in HepG2 and 4T1 cells in the presence or absence of 100 nM insulin treatment. *n* = 3 experimental repeats. (D-E) Representative images (D) and quantifications (E) of the PLA assay showing interactions between IRβ and DNAJA2 in the presence (+Ins) or absence (-Ins) of insulin. *n* = 2 experimental repeats. (F-G) Representative images (F) and quantifications (G) of IRβ subcellular intensity ratios (plasma membrane (PM), intracellular compartment (IC)) in WT, DJ2$^{-/-}$ and *DNAJA2*/*AP2B1* double KO (DKO)

4T1 cells. Scale bar, 10 μm. *n* = 2 experimental repeats. Data are shown as means ± SEM (E) or median (G). P values were determined by two-tailed unpaired t test with Welch's correction. ****p<0.0001. Source data are provided as a Source Data file.

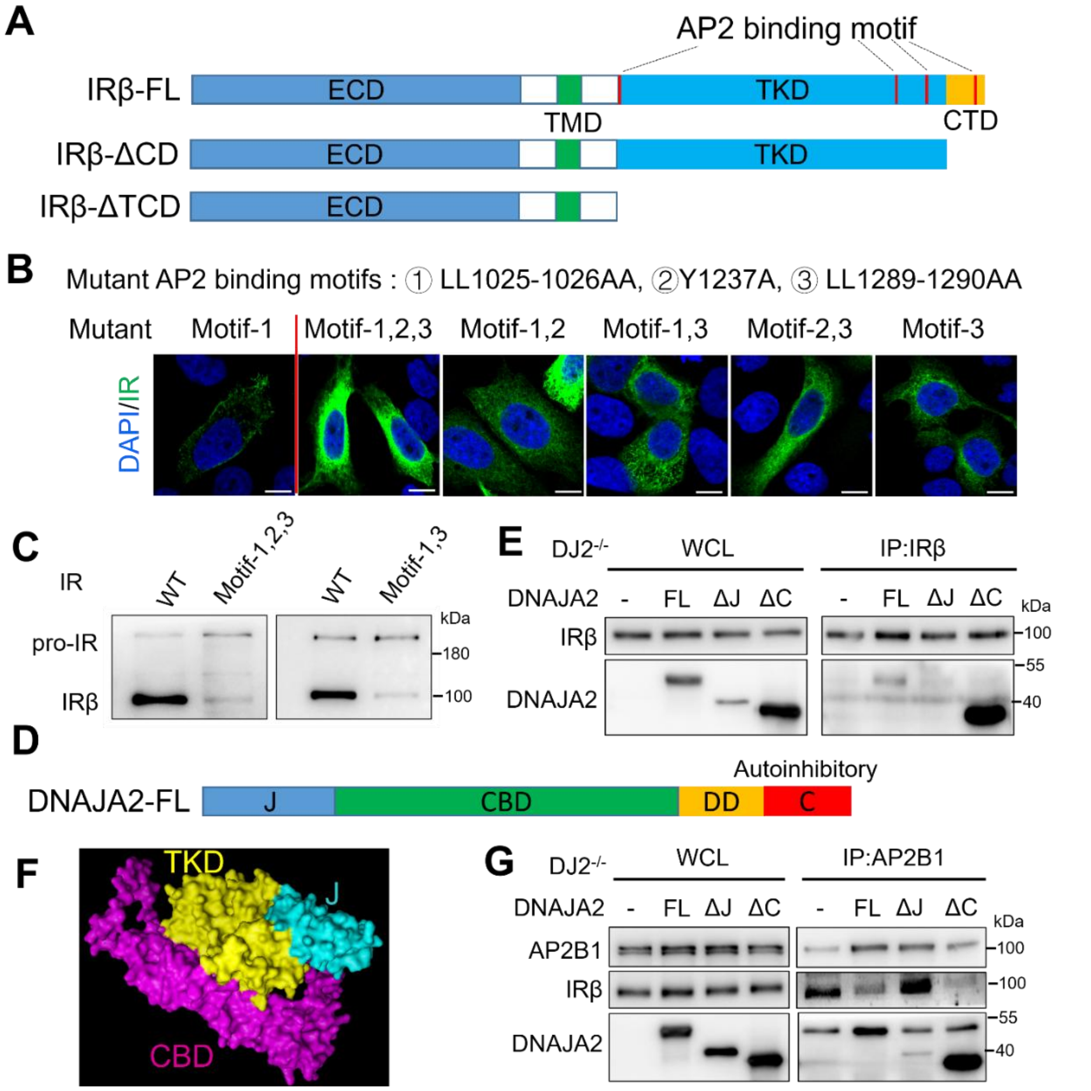

Supplementary Figure 6. The J domain of DNAJA2 facilitates IRβ-DNAJA2 interaction, related to Figure 5. (A) Schematic diagraphs showing the domains in IRβ, including extracellular domain (ECD), transmembrane domain (TMD), tyrosine kinase domain (TKD) and C-terminal domain (CTD). (B) Immunofluorescence analyses of IR subcellular localization in HeLa cells expressing IR mutants with different mutations of the potential AP2 binding motifs. (C) Immunoblot analyses of the

expression levels of pro-IR and IRβ in HeLa cells expressing WT or mutant IR, as indicated. *n* = 2 experimental repeats. (D) Schematic diagraphs showing the domains in DNAJA2, including J domain (J), client binding domain (CBD), dimerization domian (DD) and the C-terminal autoinhibitory domain (C). (E) Co-IP assay showing the interaction between IRβ and various DNAJA2 fragments in DJ2$^{-/-}$ HeLa cells ectopically expressing empty vector (-), FL DNAJA2 or DNAJA2 with deletions of J domain or C domain. *n* = 3 experimental repeats. (F) Protein structure docking analysis showing the interactions between the TKD domain in IRβ and the J and CBD domains in DNAJA2. (G) Co-IP assays showing the interaction between IRβ and AP2B1 in DJ2$^{-/-}$ HeLa cells expressing a various forms of DNAJA2 as indicated. *n* = 2 experimental repeats. Source data are provided as a Source Data file.

Supplementary Table 1

REAGENT or RESOURCE	SOURCE	IDENTIFIER
**Antibodies**		
**Insulin Receptor β (E9L5V) XP® Rabbit mAb**	Cell Signaling Technology	Cat# 23413, RRID:AB_2924796
**Phospho-IGF-I Receptor β (Tyr1135/1136)/Insulin Receptor β (Tyr1150/1151) (19H7) Rabbit mAb**	Cell Signaling Technology	Cat# 3024, RRID:AB_331253
**Phospho-Akt (Thr308) (D25E6) XP® Rabbit mAb**	Cell Signaling Technology	Cat# 13038, RRID:AB_2629447
**Akt (pan) (C67E7) Rabbit mAb**	Cell Signaling Technology	Cat# 4691, RRID:AB_915783
**GSK-3β (D5C5Z) XP® Rabbit mAb**	Cell Signaling Technology	Cat# 12456, RRID:AB_2636978
**Phospho-GSK-3β (Ser9) (D85E12) XP® Rabbit mAb**	Cell Signaling Technology	Cat# 5558, RRID:AB_10013750
**Rab7 (D95F2) XP® Rabbit mAb #9367**	Cell Signaling Technology	Cat# 9367, RRID:AB_1904103
**p-Akt1 (11E6)**	Santa Cruz Biotechnology	Cat# sc-81433, RRID:AB_1125472
**Akt1 (5C10)**	Santa Cruz Biotechnology	Cat# sc-81434, RRID:AB_1118808
**beta-Adaptin (A-5)**	Santa Cruz Biotechnology	Cat# sc-74423, RRID:AB_2058201
**Clathrin LC (CON.1)**	Santa Cruz Biotechnology	Cat# sc-12735, RRID:AB_627264
**Anti-α Tubulin Antibody (B-5-1-2)**	Santa Cruz Biotechnology	Cat# sc-23948, RRID:AB_628410
**Anti-HSC70 Antibody (B-6)**	Santa Cruz Biotechnology	Cat# sc-7298, RRID:AB_627761
**Anti-HA-Tag Antibody (F-7)**	Santa Cruz Biotechnology	Cat# sc-7392, RRID:AB_627809
**Anti-Insulin Receptor Antibody, beta subunit, clone CT-3**	Millipore Sigma	Cat# MABS65, RRID:AB_10563109
**Mouse Anti-DNAJA2 mAb, clone 2A11-F2**	Millipore Sigma	Cat# WH0010294M1, RRID:AB_1841322
**Rabbit Anti-DNAJA2 antibody [EPR11302(B)]**	Abcam	Cat# ab157216, RRID:AB_2650527
**DNAJA2 Polyclonal antibody**	Proteintech	Cat# 12236-1-AP, RRID:AB_2230709

**Na+/K+-ATPase Rabbit mAb**	Abclonal	Cat# A11683, RRID:AB_2861628
**Chemicals, Peptides, and Recombinant Proteins**		
**ProLong™ Diamond AntifadeMountant with DAPI**	Molecular Probes™	Cat# P36962
**Insulin solution human**	Millipore Sigma	Cat# I9278
**Insulin, Cy3 labeled**	NANOCS	Cat# IS1-S3-1
**Invitrogen™ Transferrin From Human Serum, Alexa Fluor™ 568 Conjugate**	Invitrogen™	Cat# T23365
**Dynasore**	MedChemExpress	Cat# HY15304
**Cycloheximide**	TargetMol	Cat# T1225
**Phosphatase inhibitor cocktail**	Beyotime	Cat# P1081
**Critical Commercial Assays**		
**jetPRIME® Transfection reagent**	PolyPlus	Cat# 114-07
**Lipofectamine™ RNAiMAX Transfection Reagent**	Invitrogen™	Cat#13778075
**Glycogen Assay Kit**	Millipore Sigma	Cat# MAK016
**Ultra Sensitive Mouse Insulin ELISA Kit**	Crystal Chem	Cat# 90080
**Glycogen Synthase (GCS) Activity Assay Kit**	Boxbio	Cat# AKSU034U
**Membrane and Cytoplasmic Protein Extraction kit**	Sangon	Cat# C510005
**Duolink® In Situ PLA® Probe Anti-Rabbit PLUS, Affinity purified Donkey anti-Rabbit IgG (H+L)**	Millipore Sigma	Cat# DUO92002
**Duolink® In Situ PLA® Probe Anti-Mouse MINUS, Affinity purified Donkey anti-Mouse IgG (H+L)**	Millipore Sigma	Cat# DUO92004
**Duolink® In Situ Detection Reagents Red**	Millipore Sigma	Cat# DUO92008
**DnaJA2 siRNA (h)**	Santa Cruz Biotechnology	Cat# sc-93101
**AP2B1 Human siRNA Oligo Duplex (Locus ID 163)**	OriGene	Cat# SR300116
**Experimental Models: Cell Lines**		
**HeLa**	ATCC	Cat# 60,005, RRID:CVCL_0030
**HeLa-*DNAJA2*$^{-/-}$**	Huang et al., 2023[1]	NA
**4T1**	ATCC	Cat# CRL-2539,

		RRID:CVCL_0125
**4T1-*DNAJA2*$^{-/-}$**	Huang et al., 2023[1]	NA
**4T1-*DNAJA2*^{OE}**	Huang et al., 2023[1]	NA
**4T1-*DNAJA2*$^{-/-}$-*AP2B1*$^{-/-}$**	This paper	NA
**MC38**	Lu et al., 2021[2]	NA
**HepG2-IR**	Choi et al., 2016[3]	NA
**Experimental Models: Organisms/Strains**		
**C57BL/6NCrl-Dnaja2em1(IMPC)Mbp/Mmucd**	Mutant Mouse Resource and Research Center (MMRRC)	Cat# 043488-UCD, RRID: MMRRC_043488-UCD
**C57BL/6-Dnaja2em1(flox)Smoc**	This manuscript	NA
**B6.Cg-Speer6-ps1<Tg(Alb-cre)21Mgn>/J**	Jackson Laboratory	Cat# 003574, RRID:IMSR_JAX:003574
**Oligonucleotides**		
**HA-IRβ forward**	5’-caagcttccttaaggacatggccaccggggcgccggcg-3’	NA
**HA-IRβ-FL reverse**	5’-TGCtctagattaAGCGTAATCTGGAACATCGTATGGGTAggaaggattggaccgaggca-3’	NA
**HA-IRβ-ΔCD reverse**	5’-TGCtctagattaAGCGTAATCTGGAACATCGTATGGGTAaaagctggggtgcaggtcgt-3’	NA
**HA-IRβ-ΔTCD reverse**	5’-TGCtctagattaAGCGTAATCTGGAACATCGTATGGGTActtctctcgagacacctccca-3’	NA
**DNAJA2-FL forward**	5’-ACGACCGGTGCCACCATGGCTAACGTGGCTGA-3’	NA
**DNAJA2-FL reverse**	5’-CGCGGATCCCTGATGGGCACACTGCACT-3’	NA
**DNAJA2-ΔJ forward**	5’-ACGACCGGTGCCACCATGGAGCAAGGTCTTCGGGAA-3’	NA

**DNAJA2-ΔC reverse**	5'-CGCGGATCCTATGTTAGGAACTTCCGGTCT-3'	NA
**Recombinant DNA**		
**pLentiCRISPR v2**	Sanjana et al., 2014[4]	Addgene Cat# 52961
**pLenti-EF1a-DNAJA2-FL**	Huang et al., 2023[1]	NA
**pLenti-EF1a-DNAJA2-ΔJ**	This paper	NA
**pLenti-EF1a-DNAJA2-ΔC**	This paper	NA
**HIR WT**	Jacob et al., 2002[5]	Addgene Cat# 24049
**HIR-HA-FL**	This paper	NA
**HIR-HA-ΔCD**	This paper	NA
**HIR-HA-ΔTCD**	This paper	NA
**HIR-HA-mut 1,2,3**	This paper	NA
**HIR-HA-mut 1,2**	This paper	NA
**HIR-HA-mut 2,3**	This paper	NA
**HIR-HA-mut 1,3**	This paper	NA
**HIR-HA-mut 1**	This paper	NA
**HIR-HA-mut 3**	This paper	NA

1. Huang, Y., Lu, C., Wang, H., Gu, L., Fu, Y. X. & Li, G. M. DNAJA2 deficiency activates cGAS-STING pathway via the induction of aberrant mitosis and chromosome instability. *Nat Commun* **14**, 5246 (2023).
2. Lu, C. *et al.* DNA Sensing in Mismatch Repair-Deficient Tumor Cells Is Essential for Anti-tumor Immunity. *Cancer Cell* **39**, 96-108.e106 (2021).
3. Choi, E., Zhang, X., Xing, C. & Yu, H. Mitotic Checkpoint Regulators Control Insulin Signaling and Metabolic Homeostasis. *Cell* **166**, 567-581 (2016).
4. Sanjana, N.E., Shalem, O. & Zhang, F. Improved vectors and genome-wide libraries for CRISPR screening. *Nat Methods* **11**, 783-784 (2014).
5. Jacob, K.K et al. Insulin receptor tyrosine kinase activity and phosphorylation of tyrosines 1162 and 1163 are required for insulin-increased prolactin gene expression. *Mol Cell Endocrinol.* **186**(1):7-16 (2002).
